# Efficacy of TAV-in-TAV using SAPIEN3 Ultra RESILIA for supra-skirtal-leakage with intravascular hemolysis

**DOI:** 10.1007/s12928-024-01007-3

**Published:** 2024-05-14

**Authors:** Ryo Otake, Daisuke Hachinohe, Ryo Horita, Juan Armando Diaz, Hidemasa Shitan, Tsutomu Fujita

**Affiliations:** Department of Cardiology, Sapporo Cardiovascular Clinic, 8-1, Kita-49 Higashi-16, Higashiku, Sapporo, 007-0849 Japan

A 79-year-old female presented with congestive heart failure secondary to severe aortic stenosis, regurgitation. Preoperative computed tomography (CT) showed a tortuous descending aorta, a horizontal aorta (Fig. 1A, B). She had a history of minimally invasive coronary artery bypass grafting. Therefore, we planned to perform transcatheter aortic valve (TAV) implantation using a 23 mm SAPIEN3 Ultra RESILIA (S3UR, Edwards Life sciences, Irvine, CA, USA), with +6% oversizing by area

**Fig. 1:**
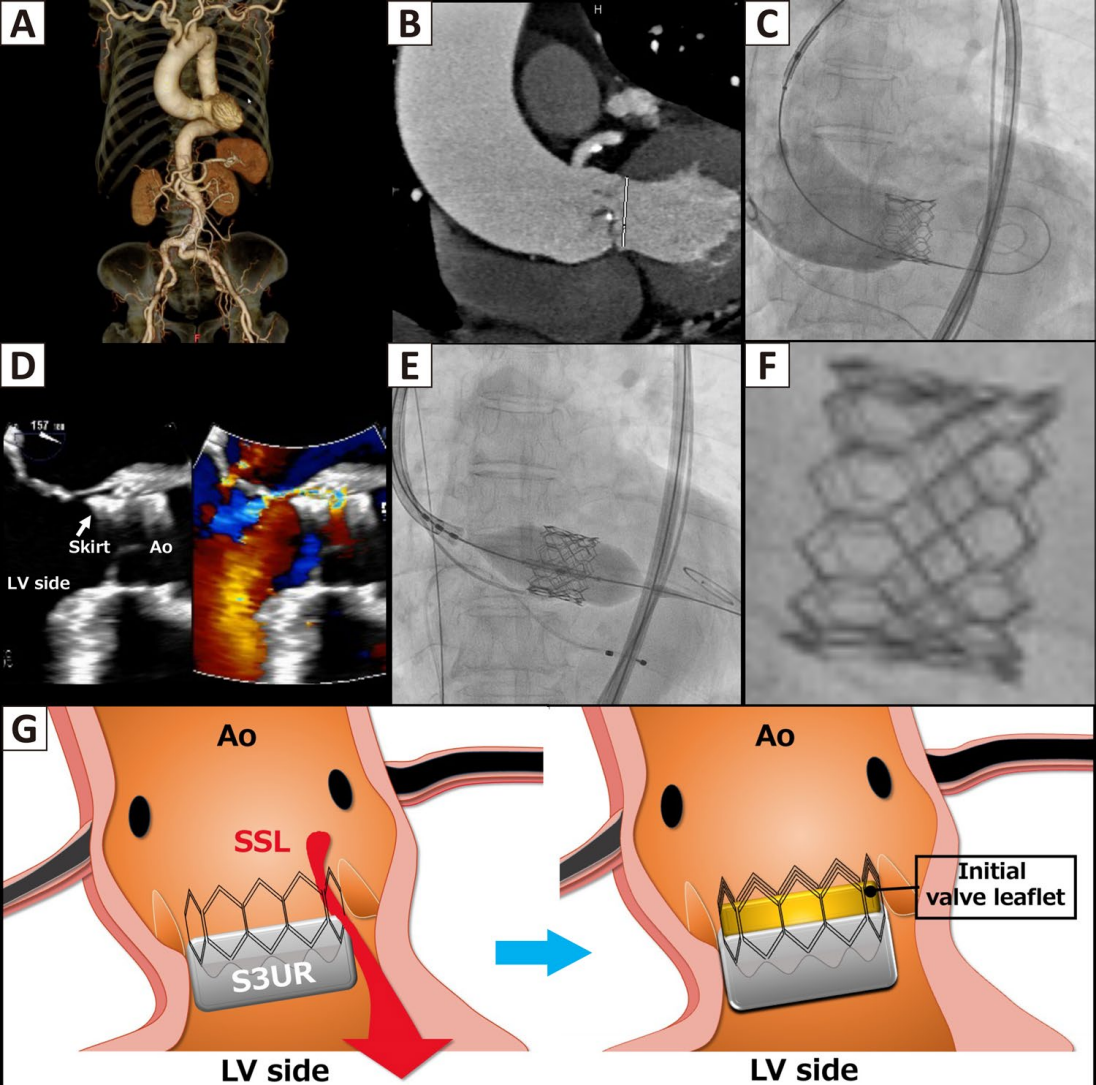
Diagnosis and Management of Supra-Skirtal-Leakage. **A**, **B** Preoperative CT. **C** Final aortography showed mild-to-moderate aortic regurgitation. **D** TEE mid-esophageal long-axis view, diastolic still-frame. **E** Procedure of TAV-in-TAV for the SSL. **F** Close-up view showing the second valve implanted slightly higher than the initial valve. **G** Schematic depiction of SSL following deep implantation of the S3UR (left) and subsequent effective sealing by the initial valve leaflet after TAV-in-TAV (right)

Unexpectedly, it was implanted too low (Fig. 1C and Online Video 1), and moderate aortic regurgitation persisted from the left coronary cusp side despite post-dilatation. Due to strong resistance, post-dilatation was performed at the same pressure, and the final transthoracic echocardiogram (TTE) showed a mean pressure gradient of 5.5 mmHg and circumferential dilatation of the valve.

Postoperatively, the patient became anemic and jaundiced. Blood tests revealed elevated indirect bilirubin levels along with decreased hemoglobin and haptoglobin findings consistent with intravascular hemolysis. Transesophageal echocardiography (TEE) revealed leakage above the prosthesis skirt, known as supra-skirtal leakage (SSL) [1] (Fig. 1D and Online Video 2). The SSL was resulted in a portion of the frame above the skirt of the initial prosthesis being positioned below the annular level; due to the progression of anemia requiring blood transfusion, a secondary procedure was required to address the SSL.

Ten days after the initial procedure, a TAV-in-TAV was performed with a second 23 mm S3UR implanted approximately 1mm above the initial valve (Fig. 1E and F). After the TAV-in-TAV procedure, the prosthesis leaflet of the first valve was raised up to the commissural level, effectively acting as a neo-inner skirt (Fig. 1G). This procedure successfully resolved the SSL and improved the hemolytic status as demonstrated by the follow-up TTE (Online Video 3) and blood test.

This case illustrates the effective use of the TAV-in-TAV procedure with the S3UR valve to address SSL. The innovative approach of using the prosthesis leaflet of the initial valve as a sealing mechanism was instrumental in resolving the SSL and associated intravascular hemolysis. This approach highlights the importance of precise-valve position and offers a potential management strategy for similar clinical cases.

## Supplementary Information

Below is the link to the electronic supplementary material.

Online Video 1. A 23mm SAPIEN3 Ultra RESILIA (S3UR) dived into the left ventricle during implantation and TAV-in-TAV using another 23mm S3UR (MP4 17371 KB)

Online Video 2. Transesophageal echocardiography Mid-esophageal Long and Short Axis Images with Color, Color Compare View, and Color 3D Image (MOV 234379 KB)

Online Video 3. Transthoracic echocardiography showing aortic regurgitation before TAVI, after TAVI, and after TAV-in-TAV (MP4 17491 KB)

## Data Availability

The data that support the findings of this study are available from the corresponding author upon reasonable request.
